# The Transcriptomics of Glucocorticoid Receptor Signaling in Developing Zebrafish

**DOI:** 10.1371/journal.pone.0080726

**Published:** 2013-11-20

**Authors:** Dinushan Nesan, Mathilakath M. Vijayan

**Affiliations:** Department of Biology, University of Waterloo, Waterloo, Ontario, Canada; Centre of Marine Sciences & University of Algarve, Portugal

## Abstract

Cortisol is the primary corticosteroid in teleosts that is released in response to stressor activation of the hypothalamus-pituitary-interrenal axis. The target tissue action of this hormone is primarily mediated by the intracellular glucocorticoid receptor (GR), a ligand-bound transcription factor. In developing zebrafish (*Danio rerio*) embryos, GR transcripts and cortisol are maternally deposited into the oocyte prior to fertilization and influence early embryogenesis. To better understand of the molecular mechanisms involved, we investigated changes in the developmental transcriptome prior to hatch, in response to morpholino oligonucleotide knockdown of GR using the Agilent zebrafish microarray platform. A total of 1313 and 836 mRNA transcripts were significantly changed at 24 and 36 hours post fertilization (hpf), respectively. Functional analysis revealed numerous developmental processes under GR regulation, including neurogenesis, eye development, skeletal and cardiac muscle formation. Together, this study underscores a critical role for glucocorticoid signaling in programming molecular events essential for zebrafish development.

## Introduction

The glucocorticoid receptor (GR) is a key mediator of the vertebrate stress response [1,2]. It is a cytosolic receptor that, after activation by binding of its primary ligand, cortisol, acts as a transcription factor to modulate gene expression, leading to energy store mobilization to cope with stress [3]. In teleosts, these actions are coordinated by the initial stressor recognition in the hypothalamus, followed by the release of corticotropin-releasing factor (CRF) that acts on the adenohypophysis, leading to release of adrenocorticotrophic hormone (ACTH) that triggers cortisol release from the interrenal tissue, which is analogous to the mammalian adrenal gland [1]. The effects of GR activation on other systems, including the immune and reproductive systems have been well characterized in a number of different model animals [4]. In teleosts, stressor recognition and a subsequent cortisol response are only present in post-hatch larvae [1,4]. 

Recent studies have revealed that cortisol and GR transcripts are maternally transferred to the oocytes and play a key role in early embryogenesis [4]. Also, studies have demonstrated novel roles for glucocorticoid signaling in early zebrafish development. GR has been linked with a variety of key developmental factors, including matrix metalloproteinases (MMPs) [5], bone morphogenetic proteins (BMPs) [6], and myogenic and cardiogenic transcription factors [6,7]. These specific molecular linkages occur alongside significant morphological deformations that result from knockdown of glucocorticoid receptor protein translation [6,8]. Perhaps most interestingly, knockdown of maternal GR alters the degradation of maternal mRNA and results in an abnormal transcriptome available for translation after the mid-blastula transition [8]. Together, these findings point to a major role for GR in development as a coordinator of a number of important embryogenic pathways.

The linkage of GR signaling to BMP expression indicates a key mechanism by which zygotic corticosteroids may influence embryogenesis. BMPs are a family of developmental morphogens that signal by binding to a number of BMP receptors, causing the activation of the SMAD family of intracellular transcription factors that then translocate into the nucleus to modulate target gene expression [9,10]. BMP signaling has been linked to numerous major developmental events, including dorsoventral patterning, mesodermal patterning, somitogenesis, myogenesis, organogenesis, and craniofacial development [9-15]. Three specific BMP genes, *bmp2a*, *bmp2b*, and *bmp4*, are modulated by GR in zebrafish [6]. 

The period of zebrafish development from 24 to 48 hpf is extremely important for early morphogenesis. This window of time immediately follows the final stages of somitogenesis [16] and includes the upregulation of muscle progenitors as myogenic differentiation increases significantly [17]. This is also a critical period of organogenesis, with the brain, pituitary, heart and vasculature, liver, gills, and interrenal tissue undergoing significant development [4,16]. By the end of this 24 h window, the embryo has a functional heartbeat and circulatory system and is ready to hatch [16], underlining the major developmental events that occur during this time. Previously, we have shown that without GR protein, zebrafish embryos cannot survive beyond 48 hpf, indicating that glucocorticoid signaling may play a role in orchestrating key developmental events [6]. 

Given the recent findings that position GR as a key mediator of developmental organization [4], we tested the hypothesis that key molecular events involved in developmental programming are regulated by GR activation in zebrafish. We specifically sought to identify novel gene regulatory networks modulated by GR signaling during zebrafish development. To this end we knocked down GR protein translation using morpholino oligonucleotides as described previously [6], and a high-density zebrafish microarray was utilized to assess the role of GR signaling on global gene expression pattern within a critical window during early development. 

## Material and Methods

### 2.1: Zebrafish care and breeding

Care and breeding of adult zebrafish was carried out as described previously [6]. Adult zebrafish were purchased from a commercial wholesaler (DAP International, Mississauga, ON) and maintained on a 14:10 light dark cycle in an AHAB recirculating system (Aquatic Habitats, Apopka, FL). Zebrafish care protocols were approved by the University of Waterloo Animal Care Committee in accordance with the Canadian Council for Animal Care guidelines.

### 2.2: Morpholino microinjection

A morpholino oligonucleotide (MO) was designed against the start site of translation for the zebrafish glucocorticoid receptor gene, and a 5 base pair mispair oligonucleotide (MP) was designed as a control; both oligonucleotides (Gene Tools, Philomath, OR) have been described and characterized previously [6]. Oligonucleotide sequences are as follows (small letters indicate altered bases in mispair control): MO: 5’-CTCCAGTCCTCCTTGATCCATTTTG-3’; MP: 5’-TGcTATgTTTAcTCTCgATACgTG-3’. Morpholino microinjection was performed as described previously [6]. Briefly, 1 nL of MO or MP was injected into the yolk of one-cell zebrafish embryos, which were reared in embryo medium [18]. Each independent sample consisted of a pool of 25 embryos that were flash-frozen at either 24 or 36 hpf. Three replicate pools of each treatment per timepoint were frozen for microarray analysis, and 5-7 pools of each treatment per timepoint were frozen for qPCR confirmation.

### 2.3: RNA extraction

RNA was extracted from pools of embryos with the RNeasy Mini Kit (Qiagen, Mississauga, ON) with DNAse (Qiagen) treatment to remove genomic DNA contamination. Preliminary RNA quantification was performed via a Nanodrop spectrophotometer (260 nm; Thermo Scientific, Waltham, MA).

### 2.4: Microarray scanning and analysis of resulting data

Microarray analysis was performed by the Vancouver Prostate Centre Microarray Facility (Vancouver, Canada). Quantification and quality analysis of RNA was confirmed by use of the Agilent 2100 Bioanalyzer (Agilent, Santa Clara, CA). Microarray analysis was carried out on three independent (each a pool of 20-25 embryos) samples each from 24 and 36 hpf embryos injected with MP or MO. 200 μg of RNA per sample was used for microarray analysis. Global gene expression in MP and MO samples was analyzed by hybridization to the Zebrafish V2 Gene Expression Microarray (Product ID 019161; Agilent, Santa Clara, CA), after one-colour labeling with the Low Input Quick Amp Labeling Kit (Agilent). Microarrays were scanned with the Agilent DNA Microarray Scanner and quantified with Agilent Feature Extraction 10.5.1.1. Data was normalized using the Agilent GeneSpring 7.3.1 software package by flooring values below 0.05 to 0.05 and normalizing data per chip to a set of positive control genes with raw data above 50 for all samples. The data was deposited to the Gene Expression Omnibus (GEO) database (GEO accession number GSE50376) run by the National Center for Biotechnology Information (NCBI). The dataset was restricted to only identified/annotated genes, omitting unidentified or hypothetical gene loci, thereby reducing the number of measured genes per sample to 12,261. The resulting dataset was then subjected to statistical and pathway analyses (see below).

### 2.5: Quantitative real-time PCR (qPCR)

Select gene expression changes were confirmed with qPCR. Embryos were injected with MO and MP as outlined above, frozen at 36 hpf, and RNA was extracted by use of the Ribozol-chloroform extraction method (Amresco, Solon, OH), following the manufacturer’s protocol. After quantification of RNA with Nanodrop spectrophotometer, 1 μg of RNA was reverse transcribed with the High Capacity cDNA Reverse Transcription Kit (Applied Biosystems, Carlsbad, CA), with the Multiscribe recombinant Moloney murine leukemia virus reverse transcriptase, producing a 20 μL mixture according to the manufacturer’s protocols. qPCR was performed as described previously [6], using 2.5 μL of cDNA in a 25 μL reaction mixture with iQ SYBR green supermix (Bio-Rad, Hercules, CA) using an iCycler iQ thermocycler (Bio-Rad). The selected genes were as follows: the BMP ligand *bmp7a*, the clotting factor *f5*, the orphaned Ftz-F1 receptor homolog *ff1d*, the structural muscle protein *myom1a*, the hormone receptor *mc1r*, and two genes involved with the stress axis and classical glucocorticoid signaling, the ACTH precursor proopiomelanocortin a (*pomca*) and the transport protein responsible for the rate-limiting step in steroidogenesis, the steroidogenic acute regulatory protein (*star*). All but one of the genes were selected because their fold-changes were statistically significant and relatively high, as well as being of interest in our exploration of the GR knockdown phenotype. Expression of only one selected gene was not statistically significant at 36 hpf, *bmp7a*, but it was chosen as it had the largest fold-change of any of the BMP ligands or receptors, and the BMP signaling pathway was considered a key target for qPCR confirmation. Primer sequences, Tm values, and amplicon sizes are provided in Table 1. Transcript abundance was normalized to β-actin as a housekeeping gene as its values did not change across samples. Normalization was performed using the ΔCT method [19] as performed previously [6].

**Table 1:** Details of primer pairs for qPCR, including forward and reverse nucleotide sequences, melting temperatures, and amplicon length.

Gene	Primer Pairs (5’-3’)	Tm (°C)	Amplicon size (bp)
*bmp7a*	F: cagggaagatgcagtggatt	62	107
	R: tcggctttggtctctcagactt		
*f5*	F: cttacaacttcccgactgtc	55	119
	R: agttggatgccacagaactg		
*ff1d*	F: gggatgatccagagaggaca	62	108
	R: cacatcggggttaaagagga		
*mc1r*	F: ctcaacagccagcgacataa	55	88
	R: caaactgatcaaaccgagca		
*mymo1a*	F: cacaaccgactcagggaaat	62	124
	R: tgcgtggctttactccttct		
*pomca*	F: gaagaggaatccgccgaaa	60	98
	R: gaagaggaatccgccgaaa		
*star*	F: tcaaattgtgtgctggcatt	60	121
	R: ccaagtgctagctccaggtc		
*ß-actin*	F: tgtccctgtatgcctctggt	60	122
	R: aagtccagacggaggatgg		

### 2.6: Statistical analyses

Statistical assessments were carried out using the R Statistical Computing environment (R Foundation, Vienna, Austria). For microarray results, the mean normalized expression was calculated for each gene at each treatment (MP or MO) and time point (24 or 36 hpf). These means were then compared within a time point using a Student’s *t*-test, followed by false-discovery rate (FDR) correction using the Benjamini-Hochberg method [20]. All p-values listed for microarray analysis are FDR-corrected. Data was not compared between time points. The complete lists of statistically significant genes at each time point are displayed in the supplemental information, along with each calculated fold-change and p-value (See Tables S1, S2). Where required, data from qPCR analysis was log-transformed to ensure normality prior to evaluation via Student’s *t*-test. For all comparisons, a value of p≤0.05 was considered significant. Functional analysis was performed with the Ingenuity Pathway Analysis (IPA) software package (Ingenuity Systems, Redwood, CA) as well as use of Gene Ontology functional annotations. An FDR-corrected p-value cutoff of p≤0.05 was used to select genes for functional and pathway analyses. Default IPA contextual analysis settings were used, including stringent filters to map direct and indirect relationships. Duplicate probes in the dataset were resolved using the minimum fold-change to maintain a conservative analysis. By the default IPA settings, 25 interactome networks were generated with a maximum of 35 genes in each network. The IPA software used the Fisher’s exact test to determine whether specific functional groupings were enriched with genes from the filtered dataset. 

## Results

### 3.1: Characteristics of microarray results

**Figure 1:**
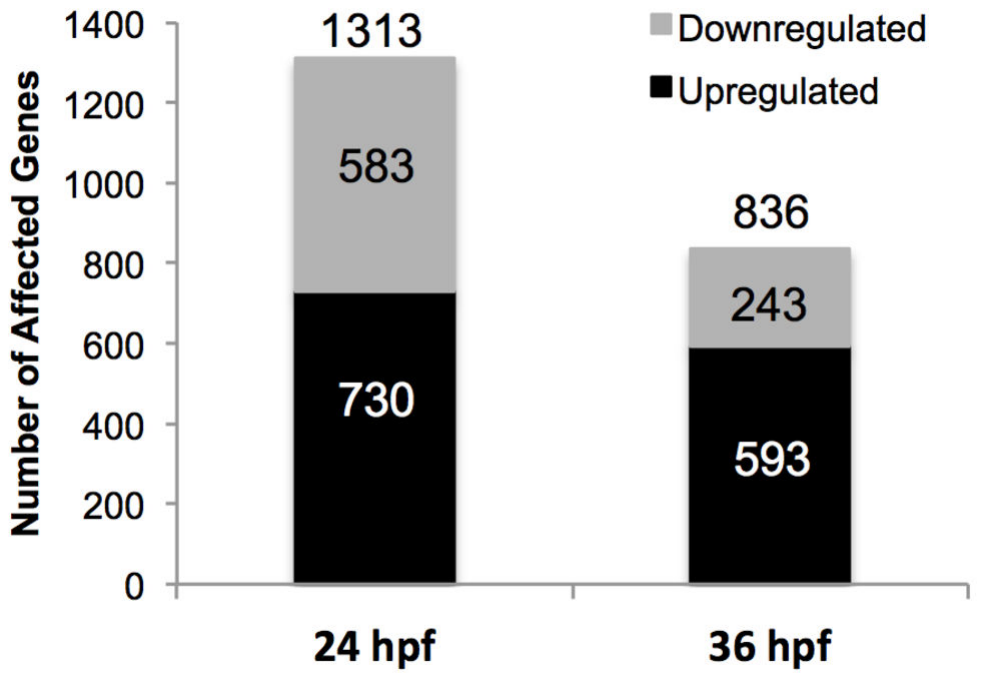
Numbers of statistically significant genes upregulated and downregulated at 24 and 36 hpf in response to GR knockdown. Of 12261 potential unique genes, the mRNA expression of 1313 were found to be statistically significantly changed at 24 hpf, with 583 downregulated (grey) and 730 upregulated (black). 836 genes were changed with statistical significance at 36 hpf, of which 243 were downregulated and 593 were upregulated. (n=3 pools of 25 embryos used for microarray analysis, P≤0.05, Students *t*-test with Benjamini-Hochberg false-discovery rate correction).

**Figure 2:**
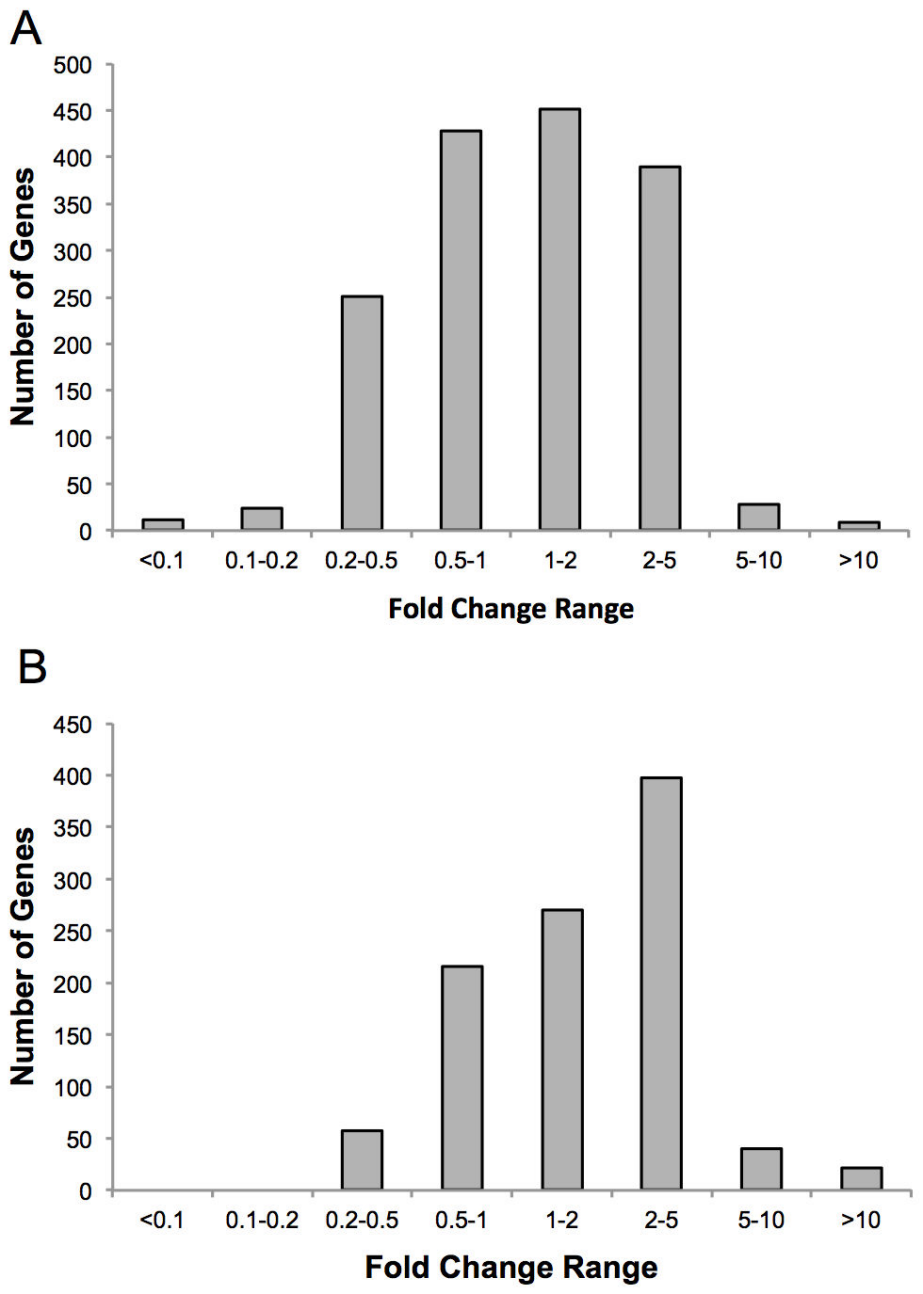
Distribution of fold-change for statistically significant genes at 24 and 36 hpf in response to GR knockdown. This figure presents the frequency of genes that were up- or down-regulated at specific fold-change ranges at 24 hpf (A) or 36 hpf (B). In general, there was a relatively normal distribution at 24 hpf, with relatively few genes showing extreme fold changes and a balance between up and downregulation. At 36 hpf, a far greater percentage of genes were upregulated than downregulated, and none showed reduction as severe as at 24 hpf.

Statistical analysis revealed that 1313 genes displayed significantly altered expression between MP- and MO-injected embryos at 24 hpf, while the expression of 836 genes was significantly different at 36 hpf. At 24 hpf, 583 of these genes were downregulated and 730 were upregulated, while at 36 hpf, 243 genes were downregulated and 593 were upregulated (See Figure 1 and Tables S1, S2). The distribution of genes along a fold-change gradient at 24 and 36 hpf is displayed in Figures 2A and 2B. The 15 genes with the highest and lowest fold-changes for each time point are displayed in Tables 2 and 3 along with their p-values, indicating genes whose transcript abundance was most affected by GR knockdown. Among the significantly changed genes at each time point, 301 were significantly altered at both 24 hpf and 36 hpf, indicating that these specific GR-modulated genes may be of particular importance in zebrafish development. Of these 301 genes, 251 were upregulated, 37 were downregulated, and 13 showed opposing changes between the timepoints (Tables S1, S2; green: upregulated; red: downregulated: blue: differential). Table 4 lists some genes that were deemed particularly interesting based on pathway analysis and gene ontology characterization of their functional grouping. These genes are organized by their general function, and listed with their fold-changes at each time point. 

**Table 2:** Select genes (top ranked based on fold-change) that were significantly upregulated in response to GR knockdown at 24 and 36 hpf, with their respective fold-change values and p-values from statistical comparisons.

**24 hpf**			**36 hpf**		
**Gene**	**Fold Change**	**P-value**	**Gene**	**Fold Change**	**P-value**
*esrrd*	49.33	0.037	*crygm2b*	45.89	0.049
*chad*	20.35	0.016	*mip2*	29.42	0.044
*mip1*	19.25	0.033	*crygm2a*	29.38	0.035
*cryba2a*	14.72	0.013	*atp2a1l*	25.43	0.045
*lim2.4*	13.74	0.041	*crygm3*	19.99	0.05
*cx44.1*	9.96	0.015	*sparcl*	15.23	0.047
*xirp2l*	8.03	0.038	*grifin*	13.89	0.032
*cryba4*	7.07	0.05	*crybb1*	13.09	0.045
*msn*	7.04	0.016	*slc25a4*	13.08	0.041
*pvalb5*	6.74	0.038	*cryba4*	11.94	0.049
*calca*	6.71	0.040	*xirp2l*	11.93	0.031
*tnnt3b*	6.42	0.039	*cryba2a*	11.49	0.034
*pvalb4*	6.35	0.011	*fabp11b*	10.87	0.05
*cpt1b*	6.29	0.009	*pfkma*	10.84	0.042
*tyrp1b*	6.18	0.018	*crygmx*	10.21	0.02

**Table 3:** Select genes (top ranked based on fold-change) that were significantly downregulated in response to GR knockdown at 24 and 36 hpf, with their respective fold-change values and p-values from statistical comparisons.

**24 hpf**			**36 hpf**		
**Gene**	**Fold Change**	**P-value**	**Gene**	**Fold Change**	**P-value**
*lect2l*	0.057	0.044	*ptgds*	0.24	0.022
*tbx16*	0.066	0.024	*trac*	0.25	0.006
*gfi1.1*	0.067	0.029	*gadd45bl*	0.26	0.020
*egln3*	0.081	0.018	*gnrh2*	0.30	0.041
*psme1*	0.084	0.040	*oc90*	0.30	0.049
*tbx24*	0.096	0.027	*flncb*	0.34	0.049
*pim1*	0.097	0.025	*gpx4a*	0.34	0.045
*tbx6*	0.098	0.037	*plekhf1*	0.35	0.047
*pcdh8*	0.104	0.045	*cryaba*	0.35	0.038
*ptgs2b*	0.104	0.015	*ddb2*	0.36	0.042
*cyp24a1l*	0.114	0.045	*hdr*	0.36	0.046
*ctsc*	0.116	0.009	*rspo1*	0.37	0.044
*ripply2*	0.121	0.042	*optc*	0.37	0.024
*cyp11a1*	0.125	0.048	*fech*	0.39	0.040

**Table 4:** Strongly-affected functional groupings (based on Ingenuity Pathway Analysis and Gene Ontology databases) with selected genes that were significantly affected by GR knockdown at both 24 and 36 hpf, with their fold-change values at each timepoint.

**Functional Grouping**	**Subgroupings: Selected Genes (24 hpf fold change, 36 hpf fold change)**
Cardiac and skeletal muscle	Cardiac ATPases: *atp2a1* (1.76, 2.59), *atp2a2a* (0.21, 0.69)
	Muscle metabolism: *ckmb* (3.03. 2.92), *pfkma* (5.91, 10.91)
	Structural proteins: *mylk3* (2.7, 3.35), *mylz2* (3.93, 2.19), *mylz3* (3.78, 4.37) *myom1a* (3.2, 2.9), *tmod4* (2.46, 2.10), *tnnc* (2.12, 1.92), *tnnt1* (1.84, 0.41), *tnnt3a* (4.75, 3.97), *tnnt3b* (6.38, 7.12), *ttna* (3.61, 2.23), *ttnb* (1.8, 2.4)
Cell adhesion, extracellular matrix	Cadherins: *cadm2a* (2.69, 2.73), *cadm4* (1.46, 1.68)
	Claudins: *cldne* (0.54, 0.50), *cldnf* (0.69, 1.61)
	Collagens: *col1a1* (2.2, 2.68), *col1a2* (1.96, 2.12), *col1a3* (3.19, 3.05), *col6a1* (2.14, 3.05), *col6a2* (2.72, 2.99)
	Connexins: *cx23* (4.15, 4.41), *cx44.1* (9.96, 2.79)
	Protocadherins: *pcdh17* (2.18, 1.97), *pcdh1a4* (2.4, 1.37), *pcdh1g18* (2.1, 1.69), *pcdh1gb2* (2.03, 1.84), *pcdh2ac* (1.93, 2.35)
	Others: *chad* (20.3, 9.65), *itgb1b.2* (3.15, 3.0)
Developmental morphogens	BMP signaling: *smad3b* (2.6, 2.96), *smad7* (0.54, 1.71)
	Hairy-related proteins: *her3* (0.56, 0.69), *her5* (0.34. 2.77)
	Others: *agr2* (2.51, 2.13), *gdf11* (1.34, 2.58),
Endocrine systems	Hormones: *avpl* (1.85, 2.75), *calca* (6.71, 2.49), *pyya* (6.15, 9.89)
	Others: *igfbp3* (1.63, 1.86), *npy1r* (2.54, 2.91), *nr5a1b* (3.18, 3.61)
Neurogenesis	Atonal homologs: *atoh2a* (2.71, 3.74), *atoh2b* (4.94, 3.00)
	LIM-domain proteins: *ldb3a* (2.2, 3.22), *ldb3b* (2.86, 3.18), *lhx1b* (1.61, 2.64), *lhx6* (3.30, 6.82)
	Others: *neurod4* (2.28, 3.27), *sox4a* (1.67, 2.732.), *sox9b* (0.39, 1.95)
Organogenesis	*mmp23a* (2.42. 2.57), *pdlim7* (2.69, 2.70), *otpb* (2.73, 2.65)
Vasculature	Clotting factors: *f5* (3.55, 2.72), *f7i* (2.46, 2.17)
	Others: *ank1* (2.95, 2.87), *vegfab* (1.76, 1.85)

### 3.2: Results of pathway analysis

**Figure 3:**
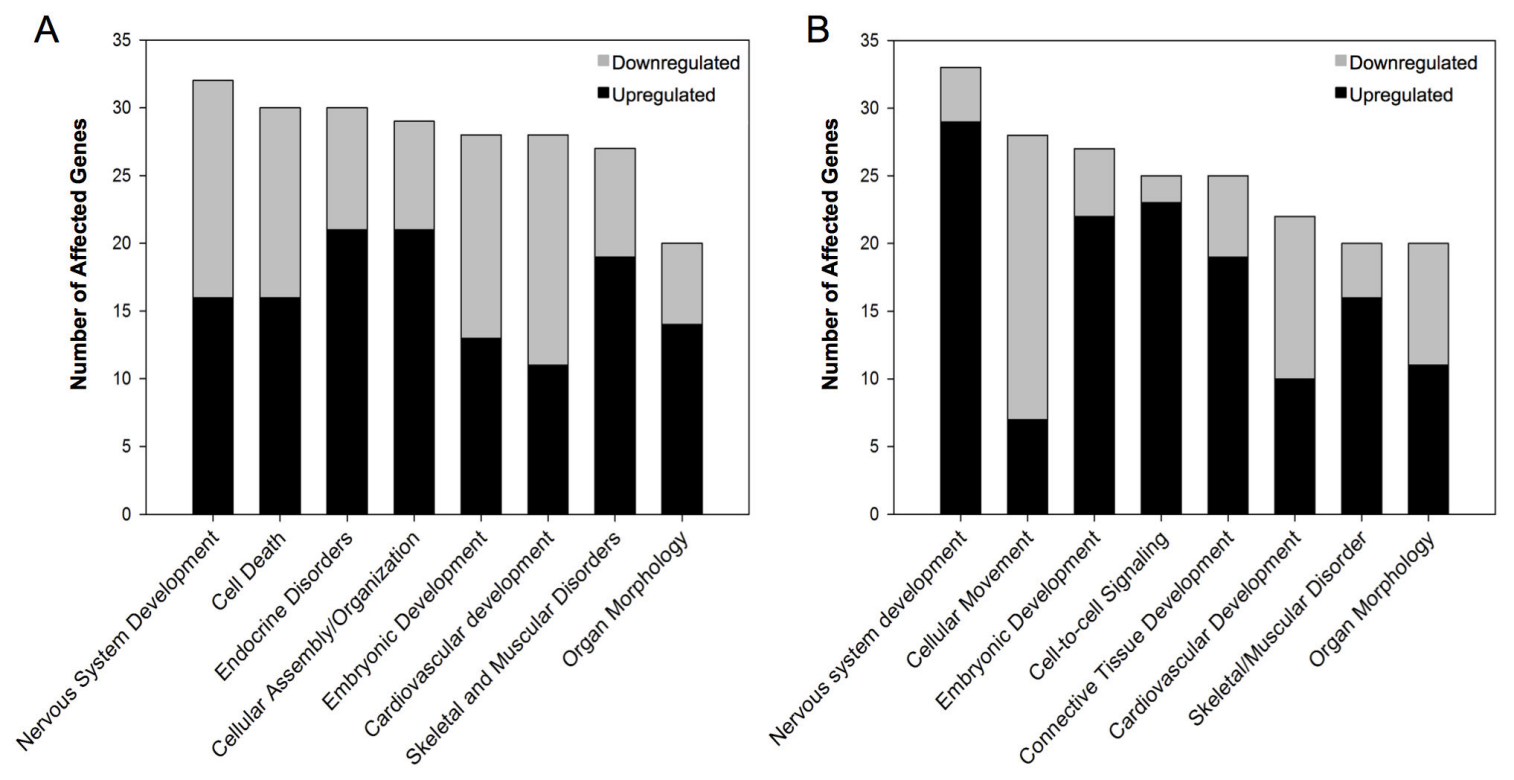
Functional annotation (using Ingenuity Pathway Analysis software) of genes that were upregulated and downregulated by GR knockdown. Ingenuity pathway analysis software identified prominent developmental pathways that were significantly affected by GR knockdown based on the significantly changed genes at 24 hpf (A) and 36 hpf (B). Each pathway is named and the total number of genes as well as the number of upregulated and downregulated genes is listed (See Tables 5 and 6 for complete list of genes).

Functional analysis using Ingenuity Pathway Analysis revealed networks of genes with known major developmental effects that were affected by GR knockdown. We have selected the top 8 networks, based on the “high score” output obtained with the IPA program reflecting a large number of affected genes in a network, and they were categorized as developmentally relevant (Figure 3). The genes and the relative numbers that were upregulated or downregulated in each network at 24 hpf (Figure 3A) and 36 hpf (Figure 3B) are provided. The genes that are grouped into these networks, the change in their expression, and their IPA scores are listed in Tables 5 (24 hpf) and 6 (36 hpf). 

**Table 5:** Gene-regulatory networks that are GR responsive as identified by Ingenuity Pathway Analysis (IPA) at 24 hpf and the calculated IPA score (higher scores denote more strongly affected pathway).

**Network**	**Genes**	**IPA Score**
Nervous system development	ascl1, calhm2, cbp(family), *cdc40, * ***cs*** *, etv4* ***,fev*** *, * ***gad1***, hdac, hes6, hes7, hes1, ***hlx1*** *, jag2* ***,klf2b*** *, krt8, mapk3, * ***mfng*** *, mpzl2, mpzl3, mtf2, * ***ndrg4*** *, notch2, notch* ***,rab38*** *, smad2, * ***smpx*** *, spry2* ***,tbr1*** *, tbx3b* ***,tph2*** *, * ***ubtf*** *, unc5b*, zeb1	45
Cell death	***adh5,aig1,aox1,aplp1,btbd2*** *, dhrs3a*, DNA-directed DNA polymerase, ***idh2,igsf21a,mthfsd,nae1*** *, piwil1, plk2, * ***pola1,pold2,ppa1,prim1***, rab5, ras homolog, *rchy1*, rho gdi, *rprm, * ***sat1***, sec 61a1, sec *61b* ***,spon1a*** *, ssr1*, tip60, *tmbim1, tp63, tp73, tp53bp2*, tsc22d1, unc119, *xpo7*	41
Endocrine disorders	***adcyap1b*** *, adora2a, arid3b, * ***camkv***, creb, dcaf13*, * ***gmps*** *, grhl1, hnf1a, hnf4a, * ***ins***, insulin**, *isoc2***, ldl, ***me3,mecr,mrps18c,nr4a2a,opa3***, penk, ***pex12,plekha8***, proinsulin, *ptpn4, * ***rab3d,rb1,rbks***, rsk, *runx1, * ***shox***, slc25a18, ***spata6*** *, ssr2, * ***syt11a,tmtc4***	40
Cellular assembly & organization	***acat1*** *, arnt2*, atpase, bves, ***calb2l,celf2,clstn1,cltcb*** *, ctssb.1, * ***entpd2a.1,irx2a,irx4a***, ldl-cholesterol, ***metap2l***, mmp, *mogat2, * ***myh6***, myh7, napg, ***nsf,plp1a***, pp1 protein complex group, *rab24*, s100a4**, *snap25***, snare, ***snx5***, stx1b, ***stxbp1***, syntaxin, ***syt4***, tcfl71, txnrd3, *vamp3, * ***zbtb33***	39
Embryonic development	14-3-3, ***afmid***, arl4a, *arntl2, * ***arrb1***, box, cbp/p300, ***cryba2a,cryba4*** *, cyp11a1, * ***epas1***, gar1, growth hormone**, *gstt1a***, hcg, histone h3, histone h4, hmga2, *hoxa10b* ***,igfbp2a,igbp3***, importin alpha, ***kpna2*** *, lef1, * ***maf***, mop10, nudt21, ***pbx1a*** *, pno1, sesn1, setd8a, shd, * ***tcf7l2*** *, tfap2a, * ***tfap2b***	37
Cardiovascular development	akap12, asf1a, *bcam*, c-src, *dusp5, egln3, eif3ea, * ***figf*** *, gata1, gata4, gata6*, gata, *gem*, hbe1, hedgehog, ***heyl,hmbsb*** *, hoxb7a, * ***htatip2*** *, jmjd6, * ***klf13l***, lama6, ***lamb2,lmo1*** *, lmo2*, mucin, ***os9***, parp, pim2, *ptpru*, secretase gamma, *sp8, tal1, * ***tal2,vegfab***	35
Skeletal and muscular disorders	***acot7,actn3a,actn3a***, alpha catenin, aph1a, ***aqp11*** *, arrdc2, * ***c*8*g***, cadherin, cdd2ap, *cdh2, * ***cdh4***, cdhe/cdhn, chmp4a, ***col6a1,col6a2*** *, dmrt2a*, dynamin, *egfr*, endophilin, ***fah***, g-actin, *invs, * ***lim2.4***, matn2, ***mdh1,parvb,pdcd6ip,pdlim7*** *, pxk, * ***sh3gl2,sparcl1*** *, thbs4b, * ***tnnt3***	35
Organ morphology	***alas2*** *, apoa1*, ck1, *cpn1*, creatine kinase, cry2, elastase, *fbln1, * ***fgb,fgf13,fgg***, fibrin, ***fibrinogen***, gpIIb-IIIa, integrin alpha V beta, mapk, *myf5, * ***myf6*** *, myog, nr6a1a, * ***pde5a*** *, per1b, * ***rgs20,scn4ab,setd3,sgcd,sgce,sgcg*** *, six4.2*, slc39a7, sox2-oct4-nanog, stat3-stat3, ***sucla2***, tni, troponin 1	23

Italicized and bold indicates significantly upregulated genes, while genes that are only italicized are downregulated.

**Figure 4:**
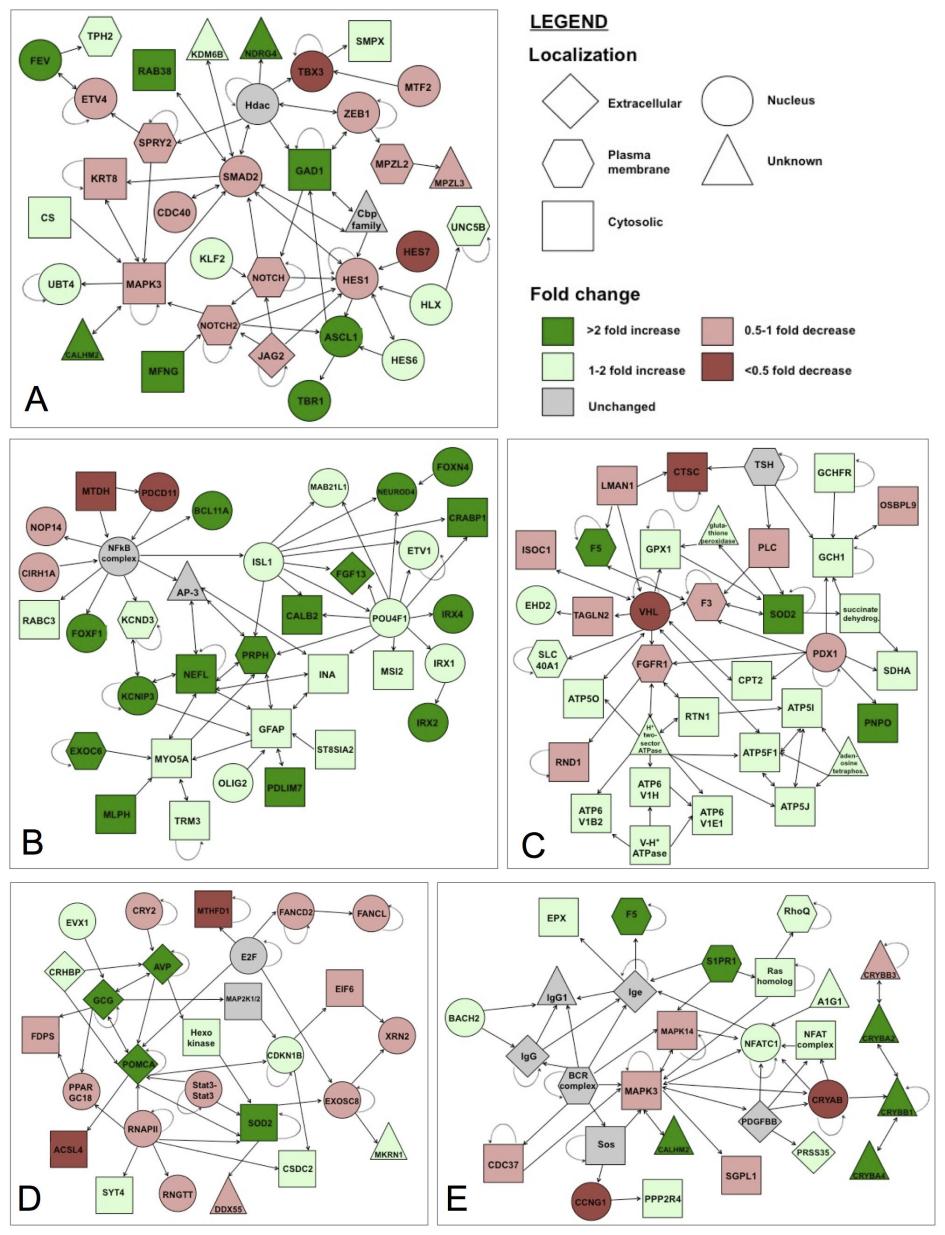
Interactome networks of select pathways identified as GR responsive by Ingenuity Pathway Analysis software. The IPA software organized and classified genes to identify important networks that were modulated by GR knockdown. These interactome networks detail the regulatory connections between genes as detailed by the connective arrows. The most strongly affected process was nervous system development at both 24 hpf (A) and 36 hpf (B). Other interesting pathways are DNA replication and energy production at 24 hpf (C), cardiovascular development at 36 hpf (D) and developmental disorders at 36 hpf (E). These final 3 pathways each involve a gene that were quantified by qPCR (Figure 4): *f5* (C, E), and *pomca* (D). Single-way arrows indicate one gene regulating another, two-sided arrows indicate co-regulation, looped arrows indicate self-regulation. The shape of each member of the network indicates its cellular location (according to IPA software classifications): Extracellular (diamond); plasma membrane (hexagon); cytosol (square); nucleus (circle); unknown (triangle). The color of each member of the network indicates its mean fold change range: >2 (dark green); 1-2 (light green); unchanged (grey); 0.5-1 (light red), <0.5 (dark red).

The IPA software also generated pathway networks that show interaction among significantly different genes and are classified according to their function. These networks were used, along with information about the cellular location and the fold change of the genes, to create interactome networks that describe cellular and whole-animal processes that appear to be strongly GR-responsive (Figure 4). At both 24 and 36 hpf, the development of the nervous system was identified as the process most strongly disrupted by GR knockdown (Figures 4A and 4B). In addition, we identified other functionally relevant processes that were identified as GR-responsive by the IPA software and that are indicative of the previously characterized GR knockdown phenotype [6]. These include transcripts involved in DNA replication and metabolic regulation at 24 hpf (Figure 4C), and cardiovascular development (Figure 4D) and developmental disorders (Figure 4E) at 36 hpf.

### 3.3: Confirmation by quantitative PCR

**Figure 5:**
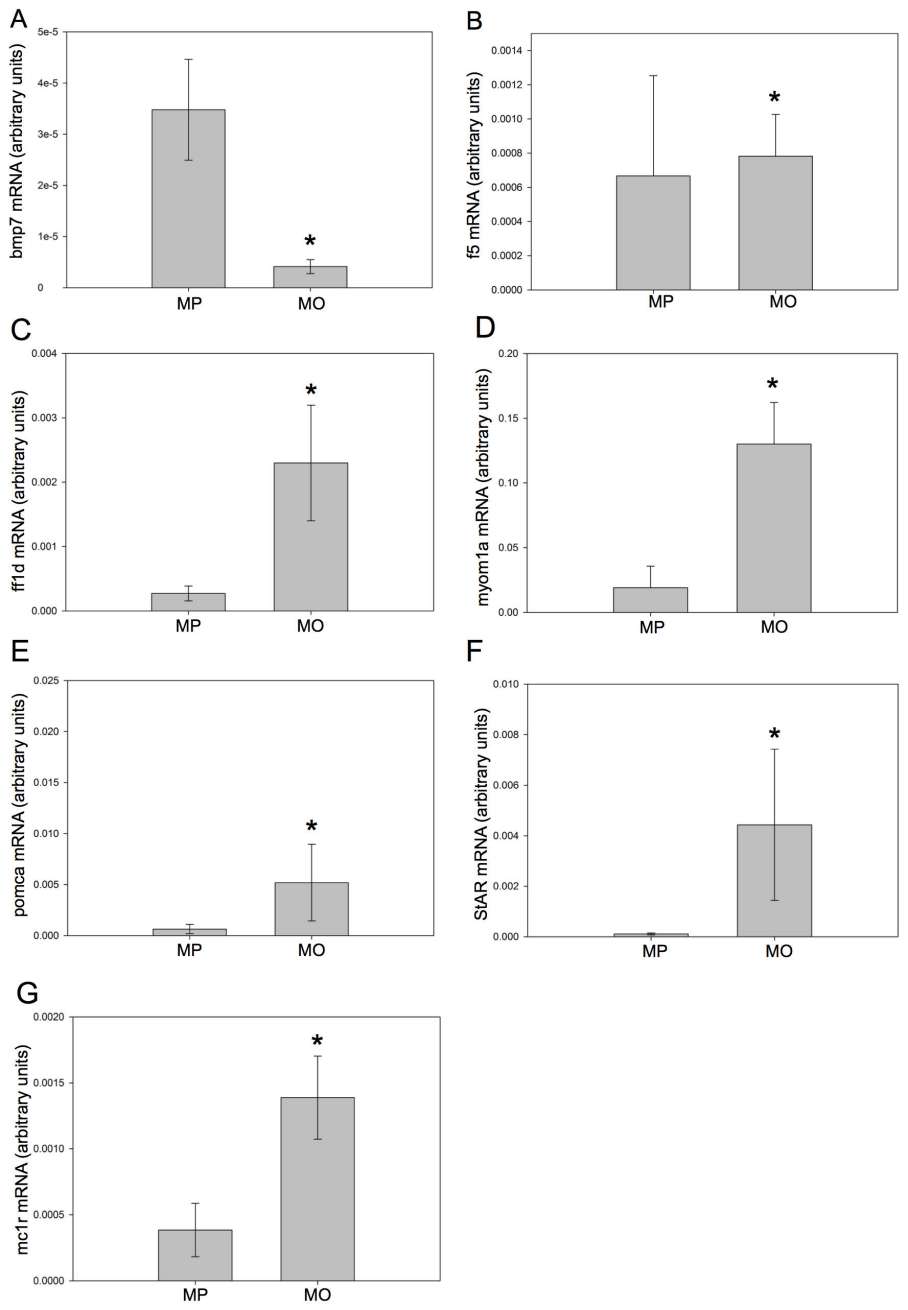
Confirmation of microarray findings by qPCR analysis. qPCR analysis was performed on 7 genes to confirm the transcript abundance seen with the microarray analysis. The selected genes were *bmp7a* (A), *f5* (B)*, ff1d* (C), *myom1a* (D), *pomca* (E), star (F), *mc1r* (G). Data is presented as mean ± standard error of the mean (normalized to β-actin, SEM; n=5-7 pools of 25 embryos each); * denotes statistical significance (*t*-test, p<0.05). (See Table 7 for fold-changes and p-values).

To confirm the reliability of the microarray results, abundance of selected transcripts at 36 hpf were quantified using qPCR (Figure 5). Genes were selected that had relatively high fold-changes, which were also deemed of potential interest in understanding the developmental changes previously observed in GR morphants [6]. These included selected genes from networks identified by IPA software as strongly affected (*f5* - Figures 4C, 4E; *pomca* - Figure 4D), as well genes identified as functionally important in development or which may be involved in the GR knockdown phenotype (*bmp7a*, *ff1d*, *mc1r, myom1a*, *star*). The qPCR analysis of gene expression was in agreement with the microarray findings, with all measured genes showing changes in the same direction and of similar magnitudes (Table 7, Figure 5). In addition, the fold-changes for all 7 genes were statistically significant with qPCR (Figure 5). This included *bmp7a*, which although having a strong reduction in fold-change (0.341), was not statistically significant in the microarray data. Table 7 shows the exact measured fold-changes and p-values for each gene examined, and Figure 5 is a graphical representation of the normalized expression, in arbitrary units. 

**Table 7:** List of genes confirmed using microarray and qPCR with fold-changes and p-values.

**Gene**	**36 hpf fold-change (microarray)**	**P-value (microarray)**	**36 hpf fold-change (qPCR)**	**P-value (qPCR)**
*bmp7a*	0.341	0.57	0.13	0.02
*f5*	1.87	0.038	1.17	0.05
*ff1d*	3.61	0.046	8.49	0.007
*mc1r*	3.72	0.04	3.61	0.020
*myom1a*	2.90	0.044	2.34	0.013
*pomca*	3.64	0.042	5.99	0.022
*star*	5.87	0.031	7.05	0.032

## Discussion

We have identified genes and pathways that are GR-responsive during early development, underscoring a key role for GR as a major developmental regulator in zebrafish [4,6]. The transcriptomics approach used in this study allowed us to further confirm some of the developmental programming events as GR-responsive based on previous studies [4,6,8], but most importantly identified several novel developmental pathways that were not previously associated with GR signaling. While there was a temporal difference in the number of significantly different transcripts in the GR morphants (higher at 24 hpf than at 36 hpf, Figure 1), a marked difference in the direction of change was also observed. The transition towards more upregulated (71%) than downregulated (29%) genes in the 36 hpf morphants points to a key role for GR signaling in the transient modulation of molecular programming events. The transient changes in overall transcript upregulation in the morphants suggest that GR activation may act as a suppressor of genes, including some key developmental transcription factors, during early development. The mechanism of GR-mediated suppression of transcription has been studied in mammalian models and appears to act via monomeric interaction of the ligand-receptor complex with other transcription factors [21,22]; however, the extent to which this occurs in teleosts remains unclear. Together with a previous study that used a high-density microarray to examine GR knockdown effects prior to MBT [8], it is becoming increasingly clear that GR actions during embryogenesis are complex but essential for development. 

### 4.1: BMP signaling pathway is GR-responsive

 We have previously indicated that the BMP signaling pathway is suppressed in response to GR knockdown [6], and the microarray results further confirm GR as a key regulator of BMP transcript levels during zebrafish development. We proposed that suppression of BMP signaling may play a role in the inhibitory effects of GR knockdown on global gene expression in zebrafish embryos, and the results of this study support this position. BMPs are important developmental morphogens that regulate developmental gene expression by activating the *smad* family of intracellular transcription factors [9]. Previously, we identified the GR-responsive BMP ligands *bmp2a*, *bmp2b*, *bmp4* [6], while this study revealed other novel members of the BMP signaling pathway, including the ligands *bmp3*, *bmp6*, *bmp7a*, and the receptors *bmpr1ab*, and *bmpr1b*. Expression of all of these genes was significantly downregulated at 24 hpf (Table S1), and this was further confirmed by quantifying *bmp7a* expression via qPCR (Figure 5A, Table 7). These results suggest that the regulation of BMP ligand and receptor expression by GR may be more widespread during zebrafish development than previously assumed. The expression of the intracellular *smad* genes, including *smad 1, smad2*, and *smad7*, that transduce signals from BMP receptor binding [9] were significantly downregulated by GR knockdown (Tables S1 and S2). Some of the *smad* genes, including *smad3a*, *smad3b*, and *smad7*, were upregulated in the GR knockdown group (Tables S1 and S2). However, these specific smad molecules have been shown to repress BMP signal transduction through *smad3* regulation of *smad7*, which has an inhibitory effect on the binding of other smad proteins to DNA [23,24]. Together, these results indicate a key role for GR signaling in the regulation of BMP-mediated functions and BMP-responsive genes during zebrafish embryogenesis. BMP signaling regulates a variety of important developmental processes, including dorsoventral patterning and initial mesodermal differentiation [9,25], as well as later morphogenic developments such as angiogenesis, myogenesis, and organogenesis [13,15]. These results lead us to hypothesize that cortisol and glucocorticoid receptor transcripts transferred from the mother may be essential for early patterning and developmental cell fate determinations, and the mechanism of action may involve modulation of BMP signaling.

### 4.2: GR-responsive pathways involved in cardiac and muscle development

In addition to BMP signaling, microarray analysis and pathway analysis identified pathways and gene interaction networks involved in skeletal/muscular disorders as being severely affected by GR knockdown (Tables 5 and 6, Figure 4). This supports recent studies showing that GR morphants displayed disrupted mesoderm formation, somitogenesis, and tail extension in GR morphant embryos [6,8]. There were several major muscle proteins among the genes that were significantly changed at both time points (Table 4), including myosins (*mylz2*, *mylz3*), troponins (*tnnt1*, *tnnt3a*, *tnnt3b*), titins (*ttna*, *ttnb*), and tropomodulin (*tmod4*). Transcript abundances of all these genes were significantly upregulated at both time points, further supporting and reinforcing the role of GR as a regulator of myogenesis [4,6]. Muscle metabolic gene expression was also affected, including genes such as phosphofructokinase (*pfkma*) and creatine kinase (*ckma*), which were upregulated at both time points (Table 4). Together these results suggest that both muscle differentiation and muscle activity are increased in response to GR knockdown, although the coordination of this activity remains unclear. Alongside the changes to skeletal muscle genes, we also saw consistent disrupted expression of cardiac muscle structural genes at both time points (Table 4), including structural fibre proteins (*mylk3*, *tnnc*), atpases (*atp2a1*, *atp2a2a*), and the cardiac transcription factor *pdlim7*. Recently we showed that exogenous cortisol administration disrupted zebrafish cardiac development, and identified some key genes, including the calcium ATPase *atp2a2a* and the cardiac transcription factor *nkx2.5*, that were downregulated in response to increased embryonic cortisol content [7]. Interestingly, these genes were also downregulated in response to GR knockdown (Tables 4, S1, and S2), leading us to hypothesize that the mode of action of abnormal zygotic cortisol content on cardiac development may involve disruption of GR signaling. Our findings in this study point to a complex regulation of cardiac development by glucocorticoid signaling, but the mechanisms involved are far from clear. It should be noted that the GR knockdown phenotype did not display cardiac edema, but there was no observable blood flow in the morphant embryos at any point in development (Nesan and Vijayan, unpublished). It is unclear whether this lack of blood flow was due to disrupted cardiogenesis or abnormal vascularization. The vascular transcription factor *vegfab* was upregulated at both 24 hpf and 36 hpf (Table 4). Knockdown of *vegfab* inhibits angiogenesis and hematopoiesis [26], but no studies have tested the effects of overexpression of *vegfab* on the developing zebrafish embryo. Our findings also show a sustained upregulation of two clotting factors, *f5* and *f7i* (Table 4), presenting a possible mechanism for the lack of blood flow in morphant embryos. If clotting factors are overexpressed, it may impede blood flow or prevent extension of developing vasculature. We further confirmed the upregulation of *f5* by qPCR (Figure 4B). A linkage between VEGF and clotting factors has been established in mammalian models [27] and while VEGF is known to induce hematopoiesis in zebrafish [26], it is unclear whether it regulates clotting factors for this action. Although angiogenesis is a complex process involving multiple pathways, the sustained upregulation of *vegfab* implies that GR signaling plays a role in modulating this critical developmental process, and may be involved the development of the circulatory system. 

**Table 6:** Gene-regulatory networks that are GR responsive, identified by IPA at 36 hpf and the calculated IPA score (higher scores denote more strongly affected pathways).

**Network**	**Genes**	**IPA Score**
Nervous system development	ap-3, ***bcl11a,calb2*** *, cirh1a, * ***crabp1,etv1,exoc6,fgf13l,foxf1,gfap***, ina, ***irx1a,irx2a,irx4a,isl1,kcnd3,kcnip3,mab21l1,mlphb,msi2b***, mtdh, ***myo5a,nefl,neurod4***, nfkβ (complex), *nop14, * ***olig2*** *, pdcd11, * ***pdlim7,pou4f1,prph,rab3c***, st9sia2, ***trim3b***	55
Cellular movement	***bmpr2b***, cofilin, ***dck*** *, ddx56, dhx16, dhx37*, DNA-directed RNA polymerase, ***eaf1*** *, ebna1bp2l, fbl, gtf2f1, * ***hnrnpm***, holo RNA polymerase II, *med19b, mybbp1a, nat10, ncl, nop56, npm1, oc90*, p70 56k, ***pdcd4b*** *, pes, polr1a, polr2b, polr2e*, ras, rnr, *rrp12*, smad1/5/8, srsf1, *ssb, * ***trpc1*** *, tsr1, wdr12*	42
Embryonic development	adrb, ***barx1***, bmp, crmp, ***dpysl2,dpysl4,dpysl5b,fbp2,gck,glud1b,gng3*** *, gng12, * ***gpc3***, hedgehog, *ihha, * ***insm1a***, jnk, l1cam, ***meis2***, nbl1, neurod1, ***nkx2.2a,pax6a,pfkb2,plp1a***, ppp2c, proinsulin, ***ptprn2,ptprn,pyya*** *,* sec *62, * ***sh3gl2***, sox2-oct4-nanog, *stam2, yes1*	40
Cell-to-cell signaling	***ache***, achr, akt, ampa receptor, cacn, ***cacna1s,cacna2d1a,cacng2***, caveolin, ***chrna4,cnih2,gnb5,gria2***, K channel, ***l-typecalciumchannel* (*cacna1s***)***,magi1*** *, musk*, n—type calcium channel, ***napg,neurod2,nptn,nrxn1b,nrxn2,nrxn3b,nsf,nxph1,rgs7bp,snap25***, snare, ***sncb***, stx1b, ***stxbp1,syntaxin* (*syn2a,syn2b***)***,vamp1,vamp2***	36
Connective tissue development	20s proteasome, 26s proteasome, ***abcc9*** *, amt, * ***arl15b,c1qtnf4,cdh10,chemokine* (*ccl-c11b***)***,clstn1,dcp1a*** *, dennd4a, * ***dnase1l3***, estrogen receptor, ***gapdh***, growth hormone, hsp70, hsp90, ***kal1a*** *, mdm2, * ***mmp24***, mmp, *nfkbiab*, nos, ***nr3c1,onecut1,pax3a,pgam1b,ret,serinc1,tbx15***, tfrc, ubiquitin, *wdr3, * ***ywhag2,znf326***	33
Cardiovascular development	*acsl4a*, avp, cdc2, cdkn18, creb, ***crhbp***, cry2, ***csdc2***, cyclin a, cyclin d, cyclin 3, *ddx55*, e2f, *eif6, * ***evx1*** *, exosc8, fancd2, fancl, fdps, * ***gcga***, got, hexokinase, map2k1/2, ***mkrn1*** *, mthfd1, * ***pomca*** *, ppargc1b*, rb, rna polymerase II, *rngtt*, rsk, ***sod2*** *, stat3-stat3, * ***syt4*** *, xrn2*	27
Skeletal/muscular disorders	14-3-3, ***acta1b,actn3a***, aldh3b1, alpha actinin, alpha catenin, ***calmodulin* (*calm1b***)***,camk2a,camk2d2,col1a1,col6a1,col6a2***, collagen type I, ***ctnna2*** *, edf1, * ***elavl4***, f actin, ***grin1b***, hsp27, itpr, ***kif23,ktn1,lrrtm1***, neurod6, pdgf (complex), pp2a, raf, rap1, *rgn, * ***smad7***, smad, smad2/3-smad4, spectrin, ***syt9b*** *, tpm3*	25
Organ morphology	*Adam17a, * ***agr2***, ampk, bhlhe41, *crlf1a*, dll1, *edn1, * ***elmo1,endothelinreceptor* (*ednrb1***)*, gtf3aa*, hdl, mlc, *mphosph10, * ***mstn***, myl4, mylpf, myosin, nadph oxidase, notch, pk, plc beta, pp1 protein complex group, pro-inflammatory cytokine, ***ptf1a***, rdx, ***rem1***, rock, *scn4aa, * ***scn4ab***, slc9a3r1, ***sp4***, tsh, ***ttn,vamp1,vegfab***	22

Italicized and bold indicates significantly upregulated genes, while genes that are only italicized are downregulated.

### 4.3: GR-responsive pathways in neurogenesis and eye formation

Another key pathway that was GR-responsive identified based on IPA interactome mapping was the development of the nervous system, which was ranked as the most strongly affected by GR knockdown (Tables 5 and 6, Figures 4A and 4B). Pathway analysis also determined that the neuron proliferation would be increased at 36 hpf. This outcome is reflected in the consistent higher expression of key genes involved in neurogenesis, including zebrafish homologs of the classical proneural gene *atonal* (*atoh2a*, *atoh2b*), at both time points (Table 4). These two genes are also known as *neurod6a* and *neurod6b*, and expression of a third neurogenic differentiation gene (*neurod4*) was also shown to be upregulated in response to GR knockdown (Table 4). All three of these genes are expressed in the zebrafish olfactory bulb [28], and play a role in vertebrate retinal patterning [29]. Together, the change in expression of these neurogenic genes indicates that GR signaling may play a role in mediating the formation of sensory apparatus and receptors. 

Retinal malformation may also occur as a result of the disrupted expression of *sox9b*, which showed a temporal expression pattern in GR morphants; downregulated at 24 hpf (Table 4, fold change 0.39) and upregulated at 36 hpf (Table 4, fold change 1.95). Numerous *sox9* targets have been identified, including cartilage and the pectoral fin, but analysis of *sox9* mutants showed a linkage to retinal neurogenesis in zebrafish [30]. The effects of GR knockdown on retinal development is of particular interest as some of the most strongly upregulated genes at both time points were lens crystalline proteins (Table 2; *cryba2a*, *cryba4*, *crygm2a*, *crygm2b*). Previous characterizations of GR morphant embryos did not reveal any noticeable effects on eye formation [6,8]. However, as the morphant embryos were only examined until 48 hpf and were developing at a slower pace [6], there may have been changes to lens or retinal formation that were not readily apparent. In zebrafish lens development, crystallins are the most abundant proteins, and are subdivided into three groupings, alpha-, beta-, and gamma-crystallins [31]. The beta-crystallins, such as *cryba2a* and *cryba4*, are expressed mainly during embryogenesis and gamma-crystallins, such as *crygm2a* and *crygm2b*, are expressed more prominently after embryogenesis in the lens of the juvenile zebrafish [32]. Alpha-crystallins tend to dominate in the mature and aging zebrafish lens [32]. The only alpha-crystallin to be significantly affected by GR knockdown, *cryaba*, was downregulated at 36 hpf (fold change 0.35, Table S2). 

The reduction of *cryaba* expression after GR knockdown indicates that it may be under direct glucocorticoid control in zebrafish, a linkage that has already been identified in mammalian cell culture models [33]. In mammals, the alpha-crystallins are expressed in various extracellular regions where they act as small heat shock proteins (HSP) to protect cells from damage due to heat and metal stress. Alpha-crystallins do not seem to accumulate outside of the lens in zebrafish [34], and although some isoforms appear to be responsive to heat-shock, their localization remains unclear [35]. To our knowledge there have been no studies linking beta- or gamma-crystallins to the cortisol stress response, so GR influence on these proteins may be associated with lens development. These results imply that lens formation and/or maturation may be a glucocorticoid-dependent process, but this requires further study.

Cell-to-cell signaling, cellular assembly, and connective tissue development were among other major pathways identified as significantly affected by GR protein knockdown by pathway analysis (Tables 5 and 6). Multiple classes of cell adhesion genes showed sustained increases in expression at both time points, including cadherins, protocadherins, and numerous collagen fibres (Table 4). Cadherins have a variety of roles in development, mainly involving the movement of cells and tissue sheets during morphogenesis [36], and the specific cadherins upregulated at both timepoints (*cadm2a* and *cadm4*, Table 4) are among those involved in nervous system development [37,38]. Protocadherins are a major subclass of cadherins, and have a variety of effects in development, with recent research showing prominent protocadherin expression in the nervous system, specifically at synaptic boundaries [39]. Multiple variants of protocadherin-1 (*pcdh1a4, pcdh1g18*, *pcdh1gb2*), a protocadherin-2 gene (*pcdh2ac*), and protocadherin-17 (*pcdh17*) were all significantly upregulated at both 24 and 36 hpf (Table 4). The roles of the protocadherin-1 and -2 genes are generally unknown in zebrafish, although there is evidence linking protocadherins to zebrafish neurogenesis, as lack of alpha-protocadherins triggers neuronal death [40]. Studies in mice have implicated protocadherin-1 in multiple developmental processes, including many that overlap with established GR effects from previous studies or what we have inferred from these microarray findings. These pathways include neurogenesis, cardiovascular development, angiogenesis, and organogenesis [41], and protocadherin-2 also affects neurogenesis in human fetal models [42]. Among the protocadherin genes with significantly affected expression in this study, protocadherin-17 is the best studied and interestingly, it is expressed prominently in the eye, and its knockdown results in reduced cell proliferation in the retina [43]. There is also further evidence for disrupted eye formation in GR morphant embryos, as the two connexin genes that are significantly upregulated at both timepoints, *cx23* and *cx44.1* (Table 4), are both expressed in the embryonic zebrafish eye [44,45]. Together, the specific cadherins, protocadherins, and connexins that are upregulated in response to GR knockdown only lend further supporting evidence to a role for glucocorticoid signaling in neurogenesis and eye formation. 

The LIM-domain proteins are another class of neurogenic genes that exhibited altered expression after GR knockdown. Three specific LIM-domain proteins (*ldb3a*, *ldb3b*, *lhx1b*), were significantly upregulated at both time points (Table 4). Two LIM domain binding proteins (*ldb3a* and *ldb3b*), are anterior CNS markers during development [46], while *lhx1b* (also known as *lim6*) is a telencephalon marker [47]. Together, the overexpression of these genes is consistent with the predicted results of the IPA functional analysis and interactome maps that point to an activation of neuronal development (Figures 4A and 4B). A second telencephalonic marker, *lhx6*, was also found to be significantly upregulated in the GR morphants at both time points (Table 4), while expression of *lhx8* showed a decrease at 24 hpf (fold change 0.76; Table S1). In addition to these telencephalon markers, the hypothalamic marker *otpb* was significantly upregulated at both timepoints (Table 4). The hypothalamus develops from the diencephalon and *otpb* is necessary for the specific development of hypothalamic dopaminergic neurons [48,49]. 

The potential involvement of glucocorticoid signaling in patterning the zebrafish hypothalamus is a very interesting finding, as the hypothalamus is the initial organizer for the stress response in vertebrates [1,2]. The dopaminergic neurons that are regulated by *otpb* produce the trophic hormones such as corticotropin-releasing hormone (CRH; or corticotropin-releasing factor, CRF, in fish) that will act on the anterior pituitary as part of the stress response and other coordinated endocrine actions [4]. Also, *otpb* is also required for the development of vasopressin-releasing cells [50]. GR knockdown increased the transcript abundance of arginine vasopressin (*avpl*), which has both osmoregulatory [51] and complex social actions [52] in mammals, but in fish appears to predominantly act to regulate social behavior in dominant/subordinate relationships [52]. This is an interesting linkage between GR knockdown and increased aggression via modified vasopressin expression in the brain. Numerous species of teleosts are known to adopt dominant/subordinate relationships within populations, and in trout this has been linked to differential activation in the stress response as measured by cortisol release [53]. Our findings in this study lead us to hypothesize that *otpb*, which is involved in development and differentiation of corticotropin-releasing factor producing dopaminergic neurons and vasopressin-expressing cells [4,50], may be active in the development of this social behavior and regulate the dominant or subordinate status of individual fish. As with previous pathways, with early embryonic GR activation only occurring via maternally deposited cortisol stores, this provides a potential role for the maternal stress state and subsequent possible alterations in cortisol deposition into the oocyte to determine the social status of offspring. 

Developmental regulation of the hypothalamus has been previously demonstrated in the ovine model where exogenous glucocorticoid exposure during early development disrupts the stress response in the mature animal by affecting circulating levels of corticotropin-releasing hormone [54]. The concept of glucocorticoid priming of the stress response is well-described in birds and reptiles, where maternal deposition into the egg is hypothesized as a stress signal that prepares the fetus for adverse conditions [55,56], but it is unknown whether this occurs in teleosts. We also observed an increased expression of other prominent genes associated with the stress response, including *pomca* and *star*, both of which were also confirmed by qPCR (Figures 5E, 5F, Table 6). The pituitary hormone ACTH is formed from *pomca* [57], and *star* is the cholesterol transport protein involved in the first step of steroidogenesis [58]. This linkage between embryonic GR signaling and hypothalamic development and stress axis functioning provides a mechanism by which maternal cortisol, which is the sole source of glucocorticoids in the pre-hatch teleost embryo [59], may regulate HPI axis development and its activity in adult zebrafish [4]. It remains unclear as to whether GR action would be directly inhibiting neurogenesis, or whether it may work via intermediate action on other organizing factors. Despite the lack of clarity in the mechanism of action, it appears that key developmental regulators of the telencephalon, the hypothalamus, and possibly other CNS regions, are modulated by GR signaling.

### 4.4: Effects of GR knockdown on endocrine signaling

The expression of other hormones, receptors, and endocrine signaling components are also significantly upregulated after GR knockdown, but their effects in the developing embryo are more difficult to assess. Calcitonin (*calca*) is the hormone produced by the ultimobranchial bodies associated with the zebrafish thyroid that regulates calcium homeostasis [60]. It was found to be upregulated at both time points (Table 4). In addition, we also observed increased expression of peptide YY (*pyya*) and the NPY receptor *npy1r* at both 24 and 36 hpf (Table 4)., Both peptide YY and NPY are involved in feeding and appetite regulation [61]. The impact of these genes in the pre-hatch, pre-feeding embryo is unclear, but if they are modulated by GR signaling, the post-hatch impact of the previously described GR priming effects on the HPI axis may control feeding patterns. We also observed a significant increase in expression of an insulin-like growth factor binding protein (*igfbp3*, Table 4). IGF binding proteins (IGFBPs) chaperone IGFs and can increase their circulating half-life [62]. IGFBP3 is the most common binding protein and the majority of IGFs are bound to IGFBP3 peptide, suggesting that *igfbp3* regulation by GR may modulate IGFs availability. This action may contribute to the reduced body size seen in the GR morphants [6]. Additionally, it should be noted that IGFBP3 has effects independent of IGF ligands, including some aspects of skeletal development [63]. Part of IGFBP3 action also results from its antagonizing effects against the BMP signaling pathway [64], and BMP1 inactivates IGFBP3 by proteolytic action [65]. While *bmp1* expression was not significantly changed in our microarray analysis, it is of interest that upregulated *igfbp3* will further suppress BMP signaling action by its inhibitory effects. Generally, between overexpression of the key hypothalamic marker *otpb* and these more disparate changes to various hormone and receptors, it appears that endocrine system function may be a prominent target for GR signaling during development. This is also supported by the pathway analysis, which lists disorders of the endocrine system as a pathway that is strongly affected by GR knockdown (Table 5).

### 4.5: Conclusions

Clearly, from the breadth of this discussion, the transcriptomics approach in response to GR knockdown has provided a wealth of information and yielded numerous areas that require further investigation. In general, this study provides a number of novel genes, pathways, and processes involved in development that are affected by GR signaling. We have found multiple major groupings of genes that support and help to explain the previously established GR knockdown phenotype [5,7,9], as well as other effects associated with GR signaling modulation in the newly fertilized zebrafish embryo, including myogenic, cardiovascular, and angiogenic disruption as well as the widespread suppression of BMP signaling molecules and receptors [5,8]. In addition, we have identified developmental pathways that have never before been associated with GR signaling, including neuronal differentiation, hypothalamic development, and eye formation. Altogether, this study underscores a key role for GR signaling in the developmental programming events, and we propose GR as a developmental morphogen essential for the development of the hypothalamus-pituitary-interrenal axis functioning in zebrafish. 

## Supporting Information

Table S1 **Full list of significantly changed genes with normalized fold-change, FDR-adjusted p-value, and identifying information for the 24 hpf time point.** Colored genes were significantly-changed at both time points: green means upregulated at both time points; red means down regulated at both time points; blue means differentially changed at each time point.
(XLSX)

Table S2 **Full list of significantly changed genes with normalized fold-change, FDR-adjusted p-value, and identifying information for the 36 hpf time point.** Colored genes were significantly-changed at both time points: green means upregulated at both time points; red means down regulated at both time points; blue means differentially changed at each time point.
(XLSX)
